# Optimization design of fiber rubber asphalt gravel sealing layer based on fatigue crack resistance test

**DOI:** 10.1371/journal.pone.0297090

**Published:** 2024-01-22

**Authors:** Jun’an Lei, Fujing Zhao, Yuanyuan Wang, Haicheng Su

**Affiliations:** 1 School of Civil Engineering and Architecture, Hubei University of Arts and Science, Xiangyang, Hubei, China; 2 School of Education, Hubei University of Arts and Science, Xiangyang, Hubei, China; 3 Xiangyang Road and Bridge Construction Group Co., Ltd., Xiangyang, Hubei, China; Shandong University of Technology, CHINA

## Abstract

Crack is one of the main diseases of pavement structure. In order to improve the anti-reflective crack ability of pavement, fiber rubber gravel sealing layer is proposed as the stress absorbing layer. In view of the shortcoming that Mcleod design method can not be associated with road performance, a sealing layer optimization design method based on fatigue crack test is proposed. Firstly, the reinforcement effect of fiber on rubber asphalt was studied through force ductility testing. Secondly, the optimum dosage of fiber, asphalt and gravel was optimized through fatigue cracking resistance test. Finally, the cracking resistance of fiber rubber gravel seal was verified through fracture energy test. The results show that fibers can significantly increase the maximum tensile force and strain yield energy of rubber asphalt, and basalt fiber has the best reinforcement effect. The most obvious effect on cracking resistance performance in the sealing layer is the amount of fiber, followed by the amount of asphalt, and finally the amount of gravel. The optimized material combination with the best crack resistance is 120g/m^2^ fiber, 14kg/m^2^ gravel and 2.4kg/m^2^ rubber asphalt, and the fatigue resistance times can reach 19532 times. The fracture energy of the composite pavement treated by the optimized sealing layer is nearly double that of the non-treated pavement structure, and it has a good anti-crack effect.

## 1 Introduction

**Fig 1:**
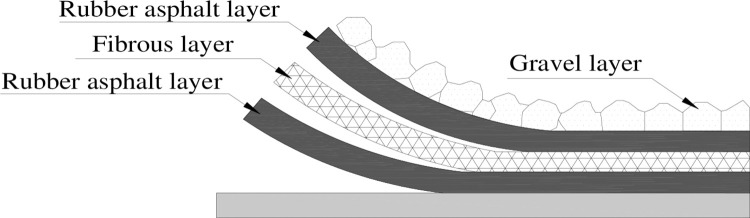
Schematic diagram of the sealing layer of fiber rubber asphalt gravel.

For pavement structures, including highway and airport pavement, cracks are one of the main diseases [1, 2]. In order to reduce reflection cracks in semi-rigid base asphalt pavement and old pavement overlay, the method of setting a stress absorption layer [3] in the pavement structure is often adopted, including geotextile [4, 5], gravel sealing layer [6, 7], flexible base layer [8, 9] and ductile concrete interlayer [10], among which the gravel sealing layer is the most commonly used stress absorption layer. The gravel sealing layer is mainly composed of asphalt and gravel. The tensile performance of asphalt at low temperatures often determines the overall crack resistance of the gravel sealing layer. Therefore, improving the low-temperature performance of asphalt is one of the effective methods to enhance the anti-reflective cracking ability of the gravel sealing layer. Rubber asphalt has good high and low temperature performance and deformation resistance [11, 12]. If it is used as a substitute for matrix asphalt, it will be beneficial for improving the performance of the gravel sealing layer [13, 14]. In addition, the existing research has shown that adding fibers to the gravel sealing layer can effectively suppress the generation of reflective cracks on the road surface [15–17]. The fiber rubber asphalt gravel sealing layer combines the advantages of both rubber asphalt and fiber [18], and its basic composition is "asphalt+fiber+asphalt+macadam", as shown in Fig 1. It can absorb the concentrated stress in the crack area, prevent the formation of reflective cracks in asphalt pavement, and have the functions of waterproofing and bonding layer, which not only improves the service life of the pavement but also reduces the maintenance costs.

In order to realize the role of fiber rubber asphalt gravel sealing layer in crack resistance and fatigue resistance, the core is the mix ratio design. In the ratio design of traditional gravel sealing layer, the amount of asphalt and crushed stone is mainly determined according to experience, or through the Mcleod theory method [19, 20], that is, assuming that the aggregate coverage rate is 100% and the average height of asphalt binder filling aggregate is 70%. The common disadvantage of empirical method and Mcleod theory method is that there is no control index. The mix ratio design cannot be linked to the pavement performance of the seal. In the design process of these two methods, the anti-reflective crack ability of the sealing layer is not considered, which is greatly influenced by subjective factors. In addition, the traditional design methods do not consider how to determine the type of fiber and the amount of fiber. Therefore, how to scientifically, accurately and reasonably design a sealing structure with good anti-reflective crack performance is very important.

## 2 Materials

### 2.1 Fiber

**Fig 2:**
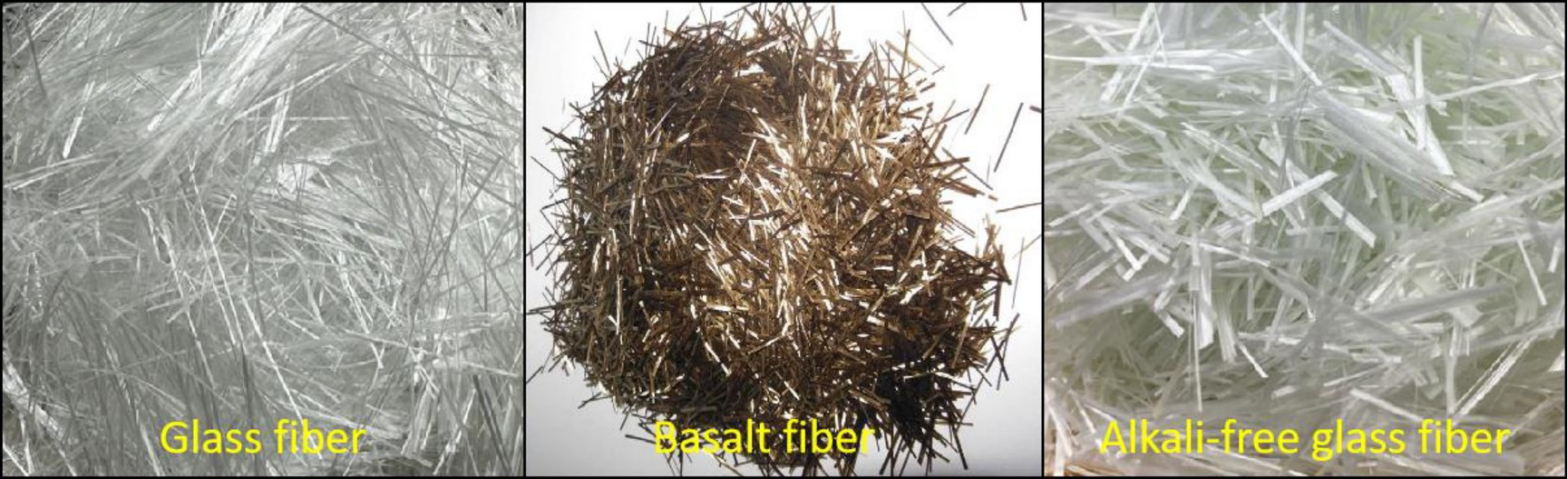
Different types of fibers.

Fiber is the core component of the sealing layer, which plays the role of strengthening, toughening, wear resistance and corrosion resistance. The fiber types selected in this study are glass fiber, basalt fiber and alkali-free glass fiber, as shown in Fig 2. The main technical indicators are shown in Table 1.

**Table 1:** Main technical indexes of fiber.

Fiber type	Monofilament tensile strength /GPa	Elastic modulus /Gpa	Elongation at break /%
Glass fiber	4.62	45.0	2.03
Basalt fiber	4.53	62.6	1.70
Alkali-free glass fiber	3.43	73.5	1.85

### 2.2 Rubber asphalt

As the main component of the stress absorbing layer, rubber asphalt can well prevent the reflection of cracks and extend the service life of the pavement. The rubber asphalt in the stress absorbing layer requires high viscosity and good elastic properties. The rubber asphalt used in this study was prepared by adding 20% of 40 mesh rubber powder into 90# matrix asphalt. The technical indicators of rubber asphalt are shown in Table 2.

**Table 2:** Technical indexes of rubber asphalt.

Index	Unit	Test value	Test method
Penetration	0.1mm	52.3	T0604
Softening point	°C	59.1	T0606
Ductility (5°C,5cm/min)	mm	124	T0605
Viscosity (177°C)	Pa.s	1.9	T0625
Elastic recovery rate (25°C)	%	70.5	T0662

### 2.3 Aggregate

The aggregate should be clean, free of weathered particles and approximately cubic. In this study, basalt aggregate with a particle size of 5 ~ 10mm was selected, and its technical indicators are shown in Table 3.

**Table 3:** Technical indexes of aggregate.

Lithology	Crush value /%	Los Angeles wear value /%	Water absorption /%	Apparent density/g/cm^3^	Relative density of gross volume
Basalt	8.5	6.6	0.54	2.928	2.882

## 3 Experimental

### 3.1 Force ductility test

**Fig 3:**
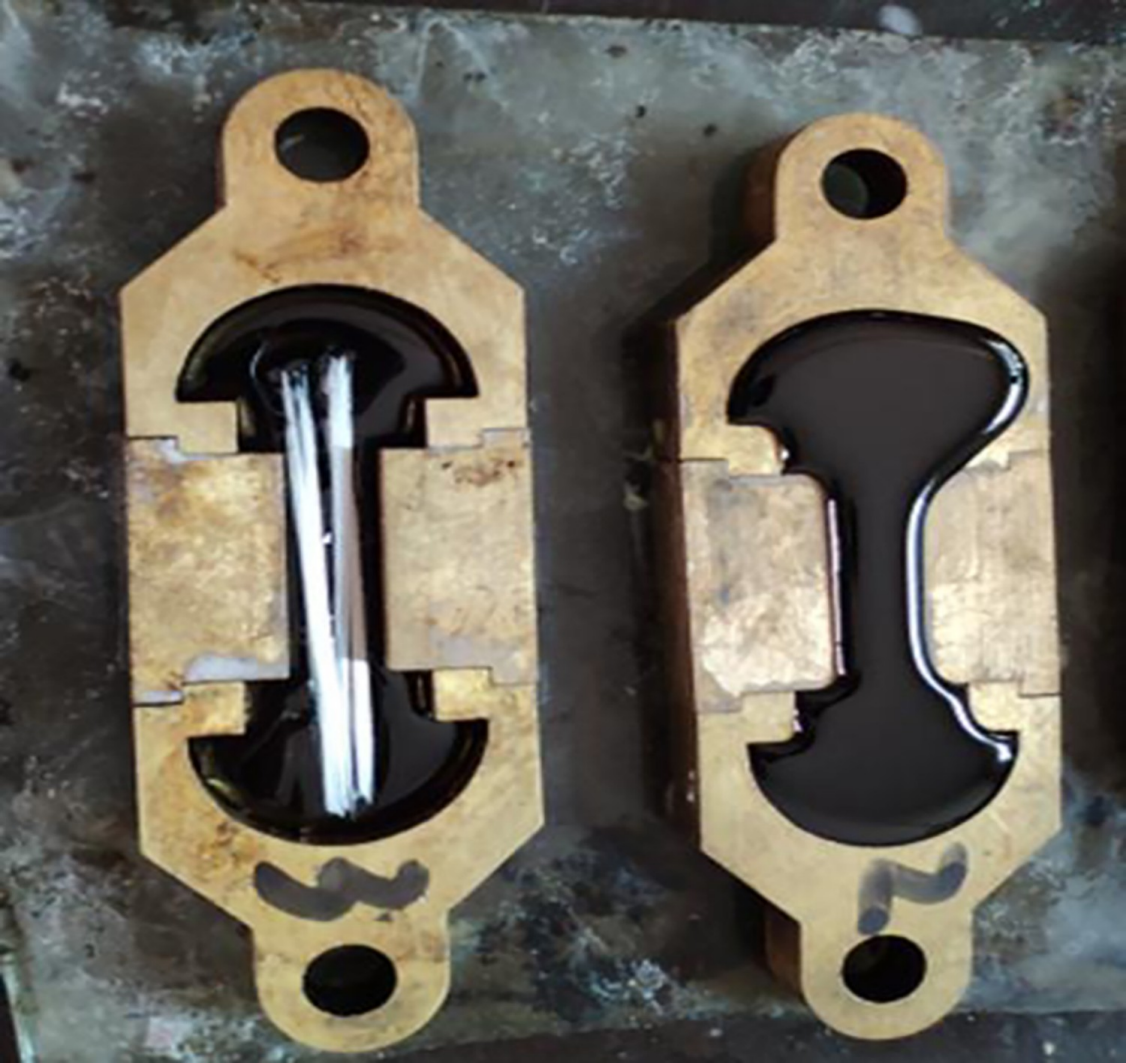
Specimen for force ductility test.

The reinforcement effect of fiber on rubber asphalt was studied by force ductility test. The specimen should be formed before the test, as shown in Fig 3. The asphalt is poured into the mold in two parts, the first is poured into half the volume of the mold, then the fiber is evenly placed on the asphalt, and finally the asphalt is poured into the mold a second time. Once the asphalt has cooled, it is scraped with a scraper. The fiber quality is 5% of asphalt, and the fiber length is 3cm, 6cm and 7.5cm, respectively.

**Fig 4:**
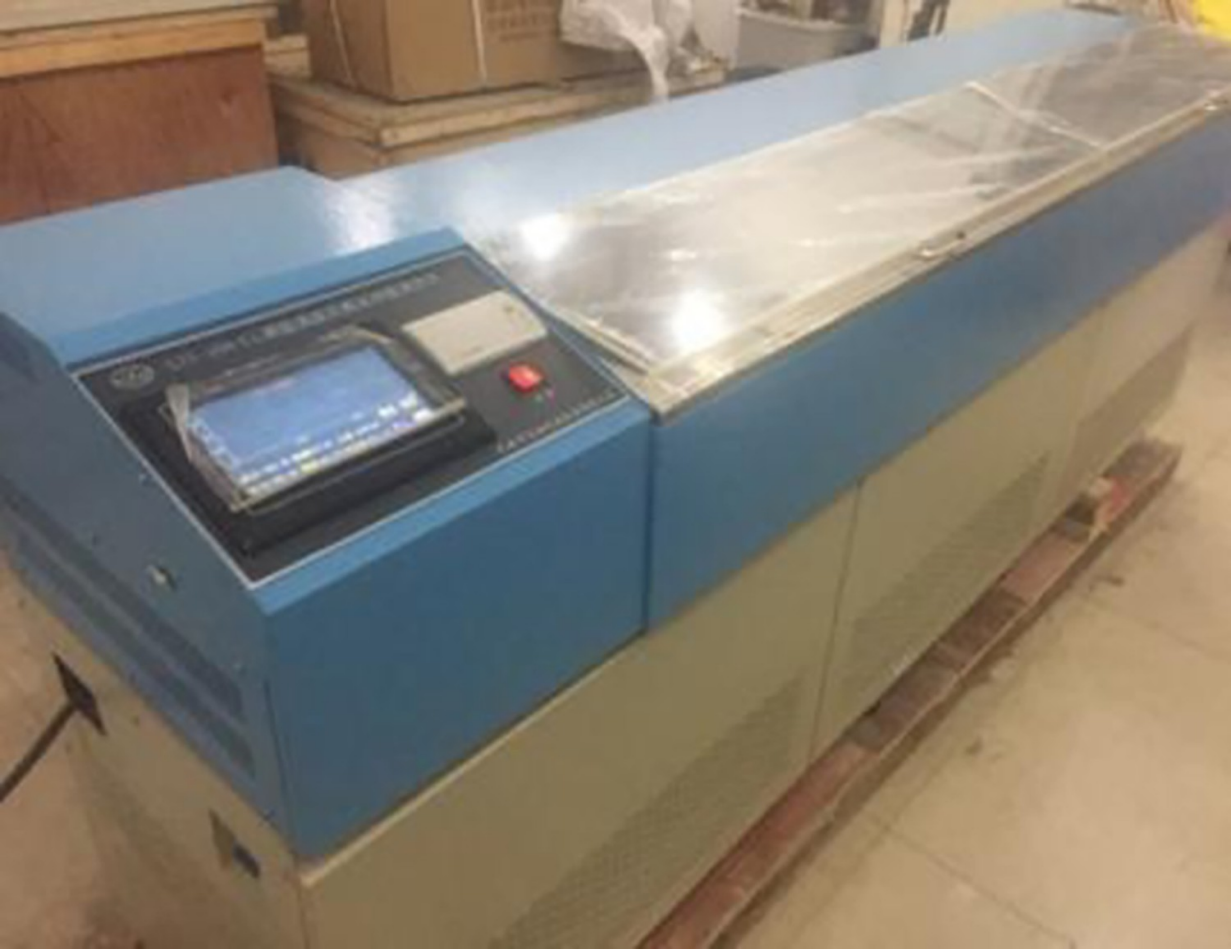
Force ductility test.

**Fig 5:**
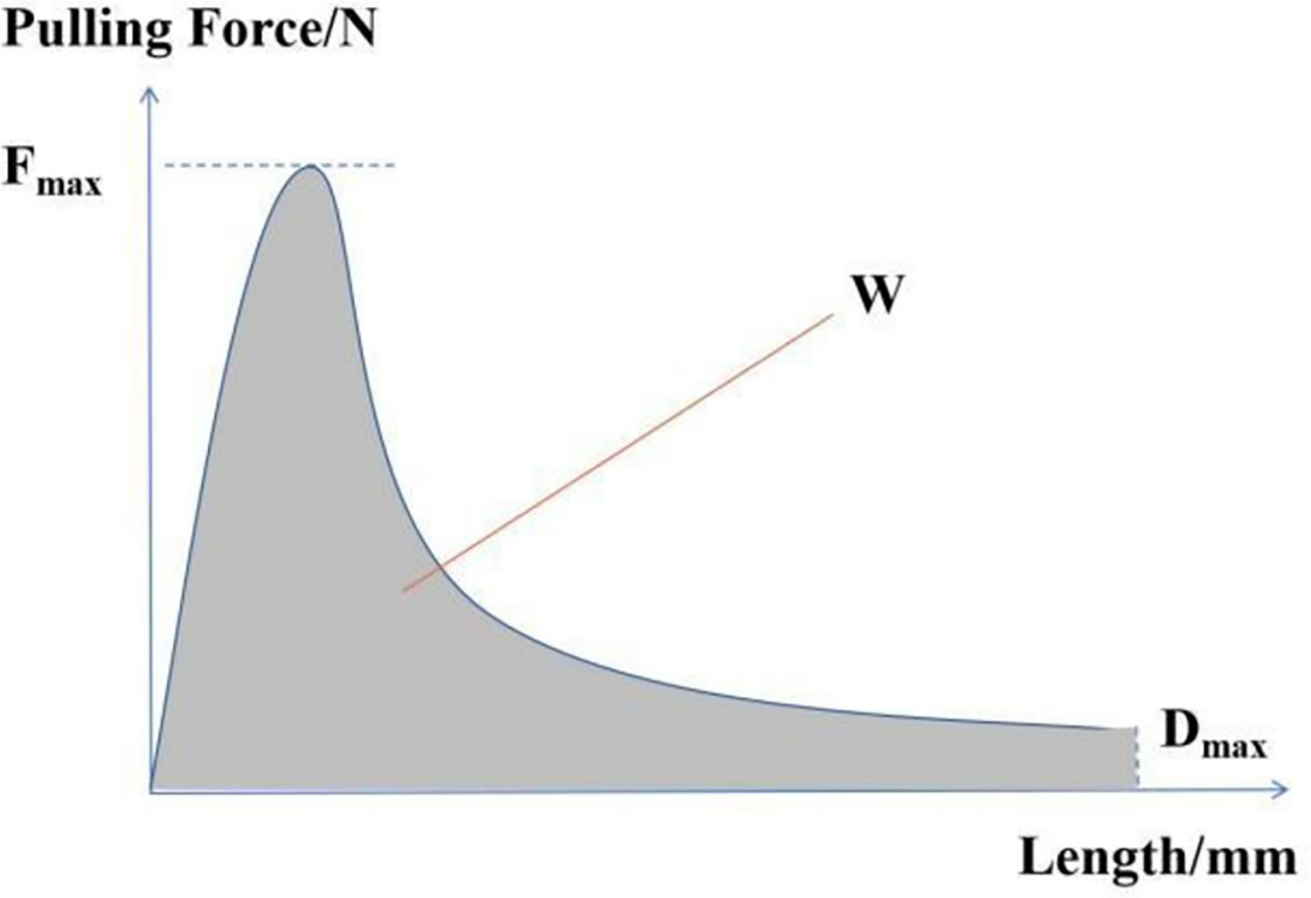
Curves of force and displacement.

The LYY-10A-CL force ductility tester produced in China was used for testing. Firstly, the specimen is installed in the instrument and immersed in 5°C water to keep warm for 1 ~ 1.5h. After that, the instrument is started to test, as shown in Fig 4. The tensile rate is 5cm/min. During the test, the data of tensile displacement and tensile force are automatically collected. Finally, the graph shown in Fig 5 is drawn according to the magnitude of displacement and force. The peak tensile force F_max_, the maximum tensile degree D_max_, and the strain yield energy W (that is, the area surrounded by the curve and the horizontal coordinate) can be calculated.

### 3.2 Fatigue cracking resistance test

**Fig 6:**
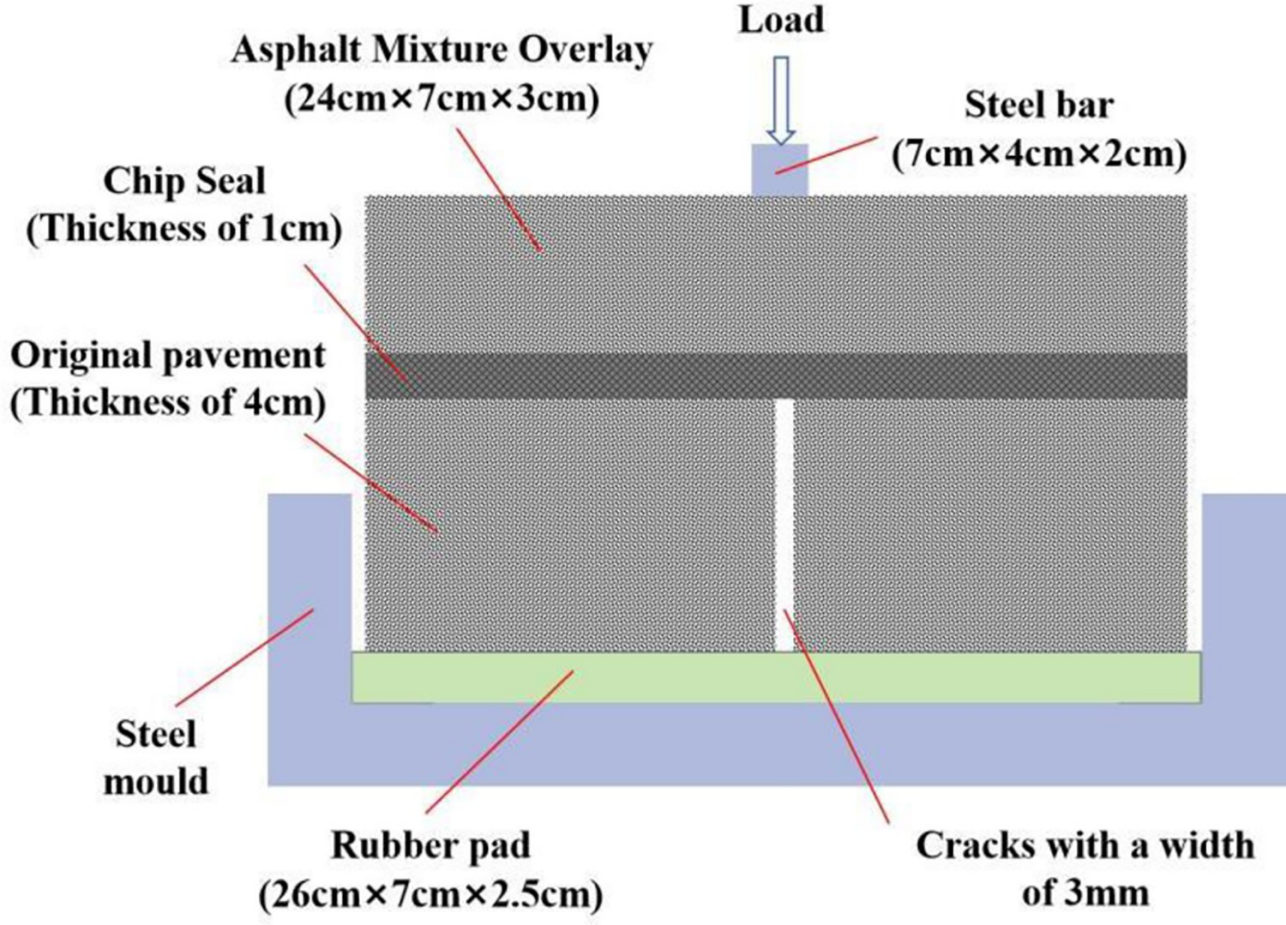
Simulation test diagram of reflection crack.

The three-layer structure shown in Fig 6 is used to simulate the anti-reflective crack ability of the fiber rubber asphalt gravel seal. The length of the specimen is 24cm, the width is 7cm, and the height is 8cm. Among them, the thickness of the original road is 4cm, the thickness of the stress absorption layer is 1cm, and the thickness of the overlay layer is 3cm. A gap with a width of 3mm is cut in the middle of the original pavement to simulate the crack of the road. The middle layer is treated with fiber rubber asphalt gravel sealing layer. The top layer is coated with asphalt mixture. During the test, the whole specimen was placed in a steel film, with a 2.5cm thick rubber block at the bottom, and fatigue load was applied at the top.

### 3.2.1 Orthogonal experimental design

The amount of fiber, asphalt and aggregate will affect the cracking resistance of the sealing layer, so orthogonal test is used to optimize the optimal ratio. The types and lengths of fibers were optimized by force ductility test, the aggregate is basalt, and the asphalt is rubber asphalt. The amount of fiber ranges from 60 to 120g/m^2^, the amount of gravel is 12 to 18kg/m^2^, and the rubber asphalt is 1.8 to 2.4kg/m^2^. Fatigue life (the number of load times when the crack completely reflects to the surface) is used as the index to obtain the best dosage of each material. Four levels were selected for each influencing factor, and orthogonal tests were carried out according to the orthogonal test table of L16(45). The orthogonal test scheme is shown in Table 4.

**Table 4:** Orthogonal test table.

Test number	Factor		
	A-Amount of fiber (g/m^2^)	B-Amount of gravel (kg/m^2^)	C-Amount of rubber asphalt (kg/m^2^)
1	60	12	1.8
2	60	14	2.0
3	60	16	2.2
4	60	18	2.4
5	80	12	2.0
6	80	14	1.8
7	80	16	2.4
8	80	18	2.2
9	100	12	2.2
10	100	14	2.4
11	100	16	1.8
12	100	18	2.0
13	120	12	2.4
14	120	14	2.2
15	120	16	2.0
16	120	18	1.8

The range analysis method is adopted to analyze the test results. The range of each factor is calculated, and then the primary and secondary relationship of influencing factors is determined by the size of the range. The larger the range, the greater the influence of the factor on the test index. The formula for calculating the range is as follows:

$$R=\max\{K_{1},K_{2},K_{3}\}-\min\{K_{1},K_{2},K_{3}\}$$ (1)

Where: R—range; K_i_—is the sum of the test indices corresponding to level i for either factor.

#### 3.2.2 Preparation of specimen

**Fig 7:**
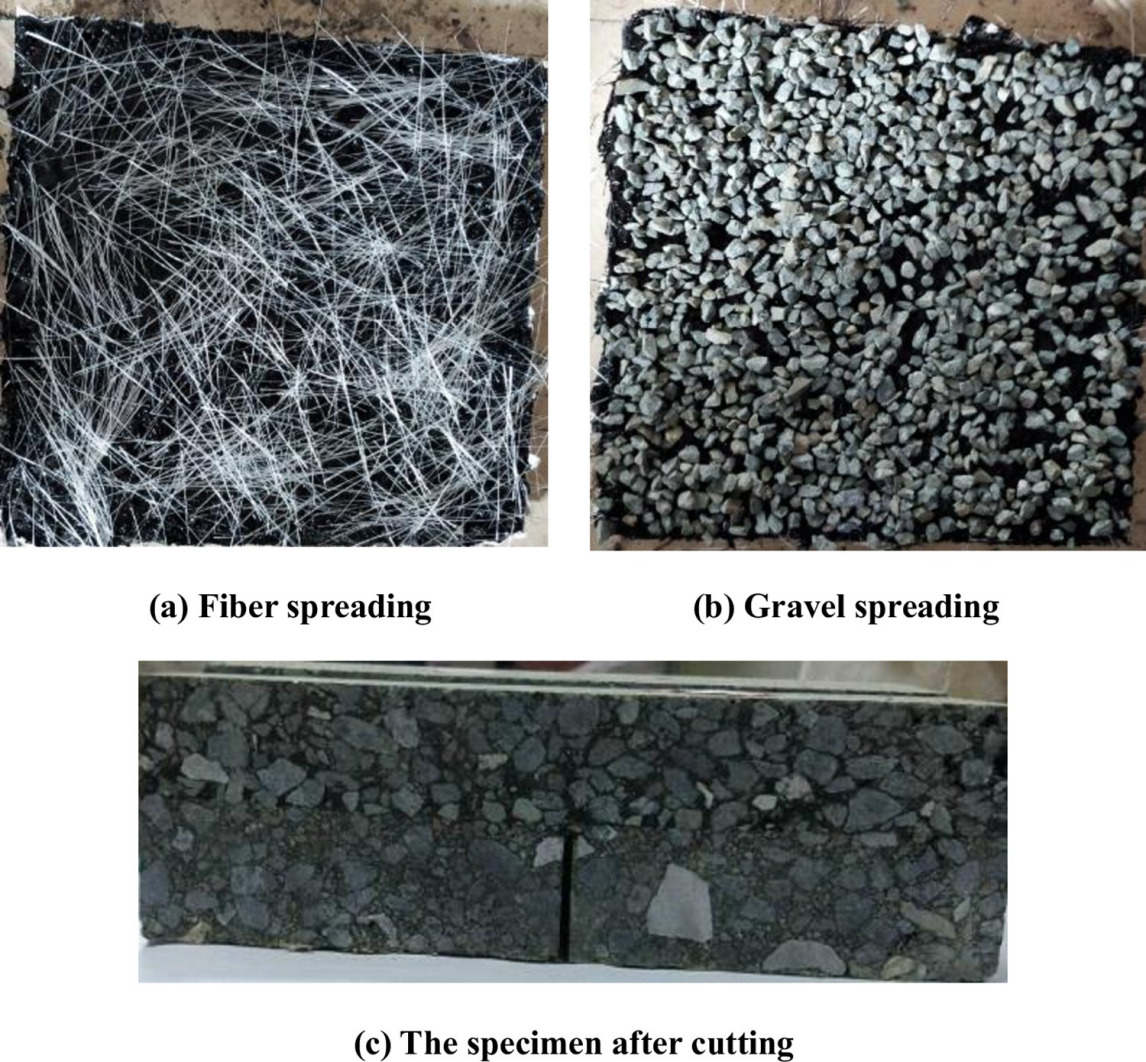
Production process of the specimen.

First, the asphalt mixture base plate with the size of 30cm×30cm×4cm was formed by a roller. Then the fiber rubber asphalt gravel sealing layer was made on the mixture according to the ratio of orthogonal test design. The sealing layer of "rubber asphalt + fiber + rubber asphalt + gravel" is made by layering. During the preparation process, the uniformity of the fiber, asphalt and gravel was ensured as far as possible, and the compaction was made, As shown in Fig 7(A), 7(B). After that, the sealing layer is coated with hot asphalt mixture with thickness of 5cm and compacted. Finally, the specimen was cut into a small specimen with a size of 25cm×7cm×8cm, and a crack with a width of 3mm was cut in the middle of the bottom of the specimen, as shown in Fig 7(C).

#### 3.2.3 Fatigue crack test

**Fig 8:**
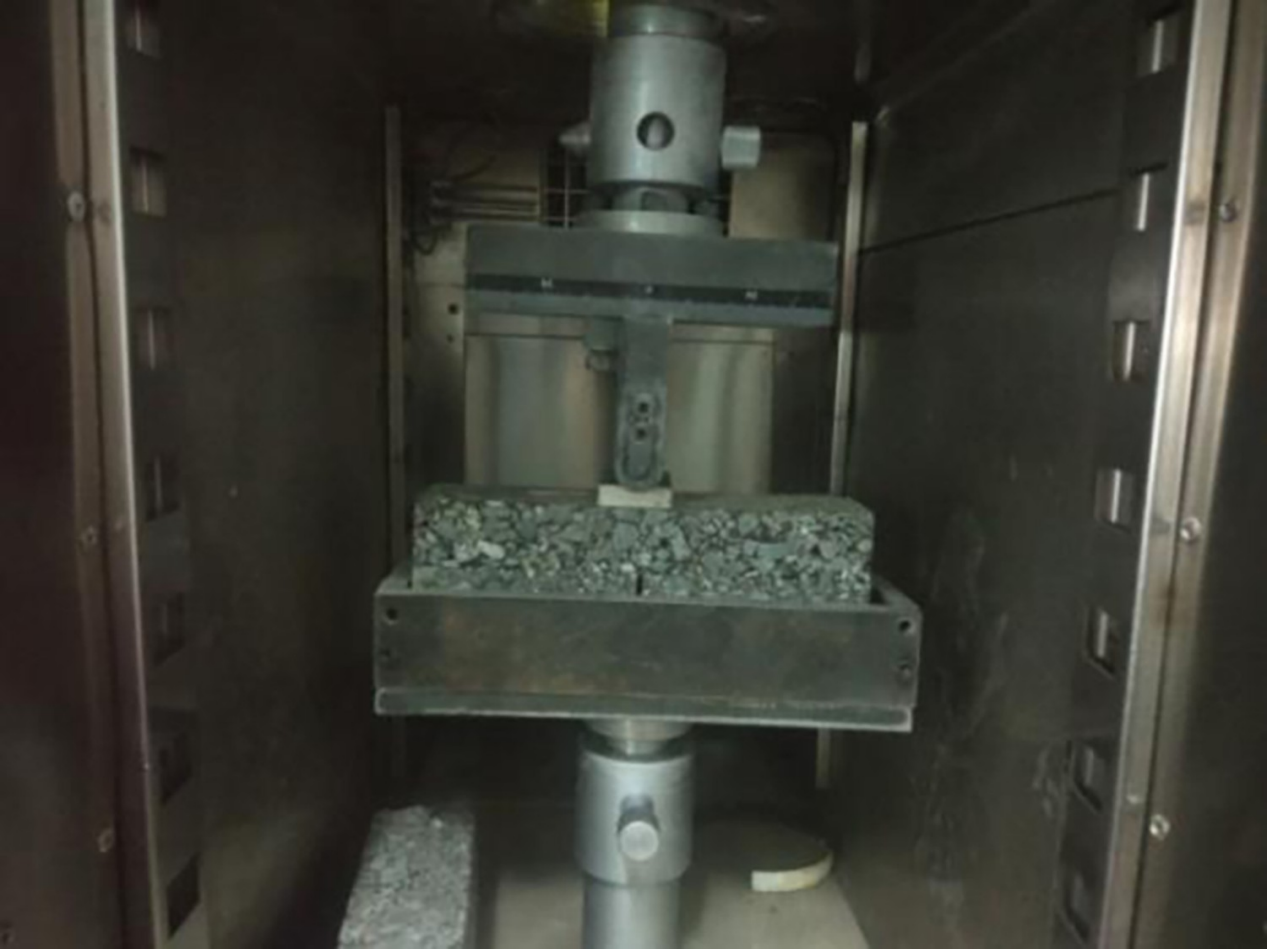
Fatigue test.

**Fig 9:**
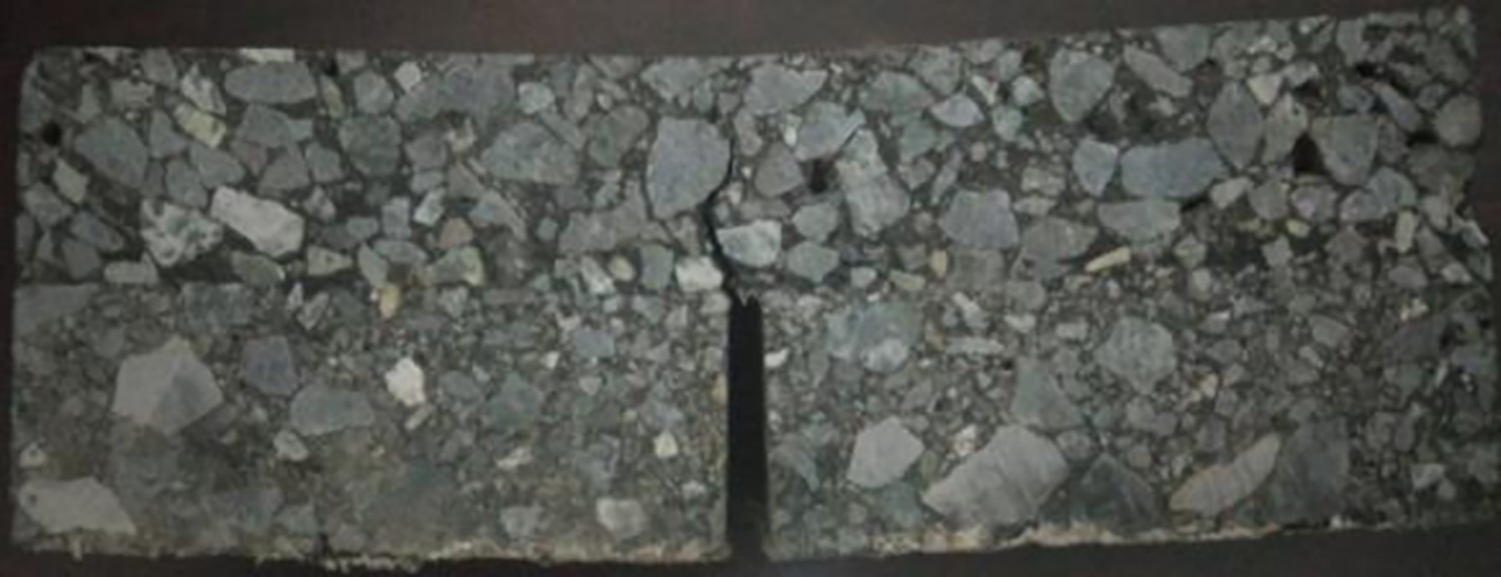
Fatigue cracking of specimen.

In this study, a universal testing machine was used to test the fatigue cracking resistance of the sealing layer. Firstly, the sample was placed in a self-made steel mold, and a rubber pad with a thickness of 2.5cm was placed at the bottom of the mold. Then, the specimen was kept in an incubator with a temperature of 15°C for 1h. Finally, the loading test was carried out after the test parameters were set, as shown in Fig 8. During the test, a strip load was applied on the top of the specimen, and the peak value of the load was 4KN. The loading waveform was half-wave sine, the loading frequency was 10Hz, and the loading area was 4cm×7cm (corresponding to 1.4MPa tire pressure). The development of cracks was observed during the experiment, and the fatigue life was recorded by recording the load times corresponding to the complete cracking of the overlay layer, as shown in Fig 9. There are at least two specimens in parallel test. If the results of the two specimens are very different, a new group is made, and the average value of the two samples with similar values is taken as the final result.

### 3.3 Fracture energy test

**Fig 10:**
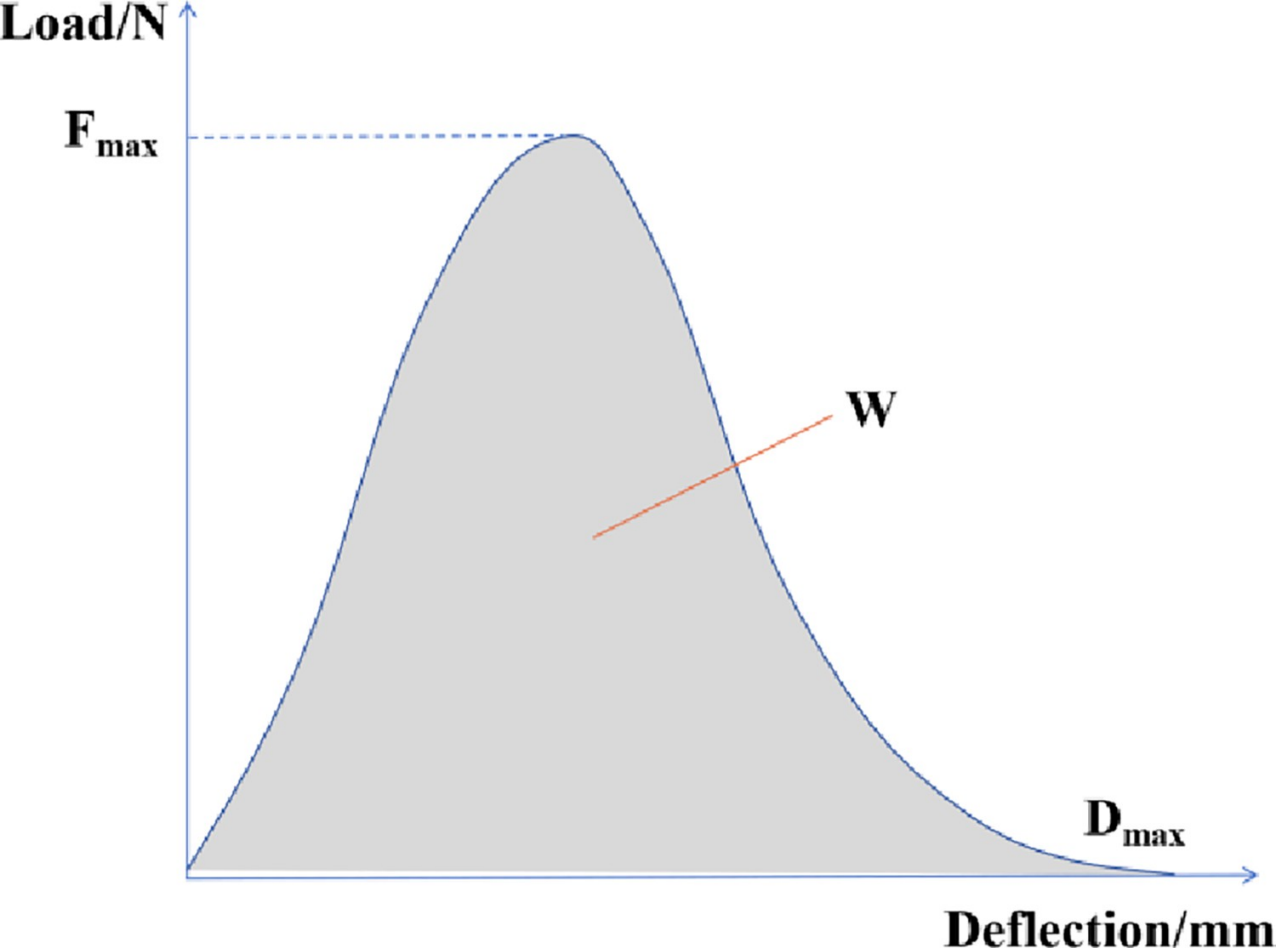
Loading curve of fracture energy test.

**Fig 11:**
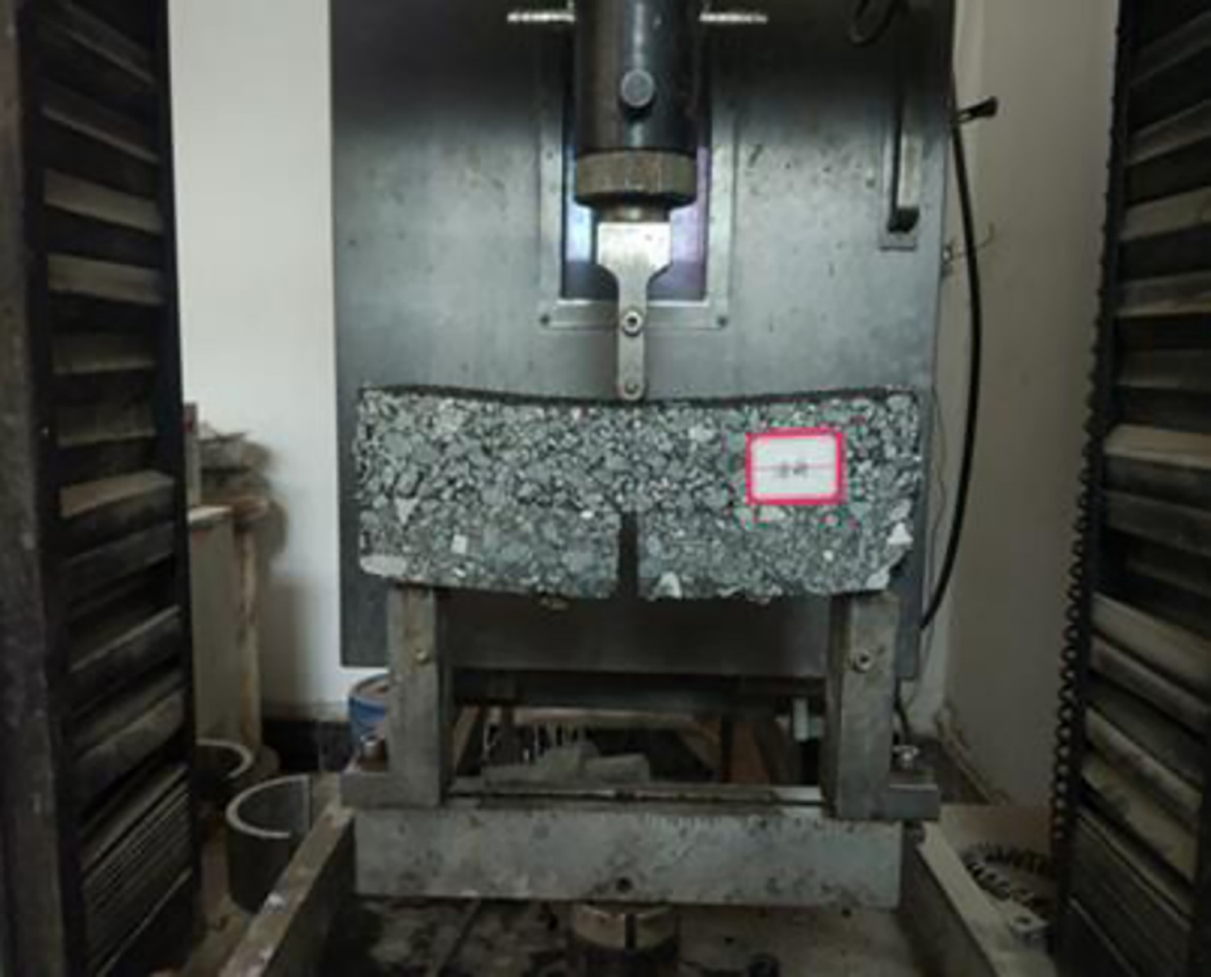
Three-point bending test.

The fracture energy refers to the energy required for the unit area of crack expansion under tensile load. It value reflects the difficulty of crack propagation. In the fracture energy test, an initial crack should be prefabricated on the specimen to ensure that the specimen expands from the initial prefabricated crack. In this study, a three-point bending test method was used to measure the load-deflection curve of the pre-cut beam under external load, and the fracture energy was determined according to the area enclosed by the curve and the coordinate axis, as shown in Fig 10. Before the test, specimens should be made. The specimens were divided into two groups, one group was equipped with optimized fiber rubber asphalt gravel sealing layer, and the other group was the control group without sealing layer. The production method is the same as the fatigue cracking resistance test. A composite plate with a size of 30cm×30cm×10cm was first formed, and then the plate was cut into a beam specimen with a size of 30cm×10cm×10cm, and a 3mm slit was reserved in the center of the beam bottom. During the test, the specimen should be kept warm at 15°C for 1h, and vertical loads should be applied in the span, as shown in Fig 11. The loading was carried out in displacement control mode. The loading speed was 2mm/min, the loading span was 250mm, and the loading termination condition was that the load decayed to 95% of the breaking load. The load and deflection can be automatically collected during the test.

## 4 Results and discussion

### 4.1 Force ductility test

**Fig 12:**
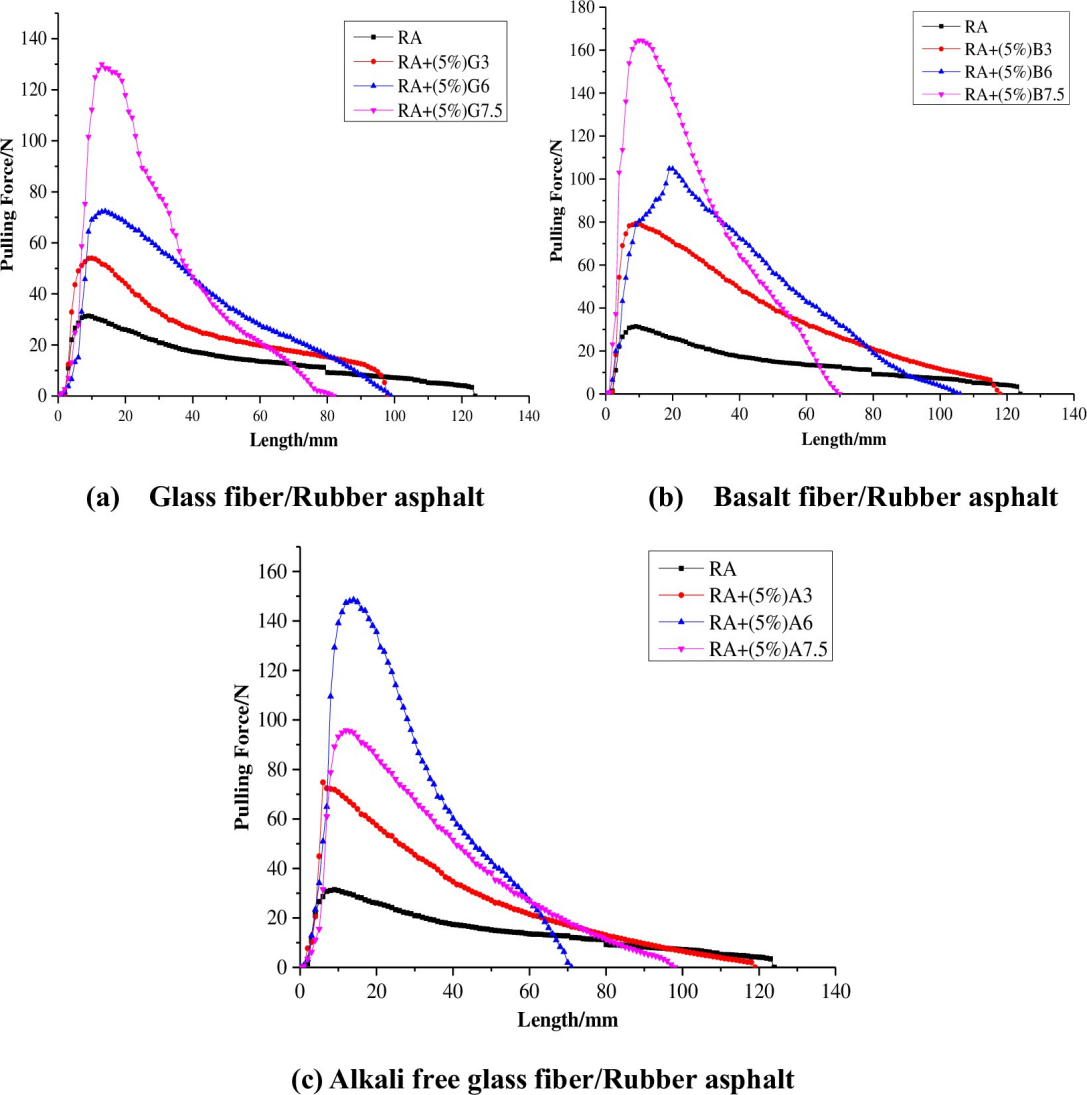
Tensile ductility of fiber rubber asphalt.

Note: RA represents rubber asphalt, 5% represents the mass percentage of the fiber in the asphalt, G represents glass fiber, B represents basalt fiber, A represents alkali-free glass fiber, and numbers represent fiber length.

The force-ductility curves of different types and lengths of fiber reinforced rubber asphalt are shown in Fig 12.

The results of maximum tensile force F_max_, maximum tensile length D_max_ and strain yield energy W calculated according to Fig 12 are shown in Table 5.

**Table 5:** Results of force ductility test.

Fiber length/cm	Glass fiber			Alkali free glass fiber			Basalt fiber		
	Fmax/N	Dmax/mm	W/N·mm	Fmax/N	Dmax/mm	W/N·mm	Fmax/N	Dmax/mm	W/N·mm
0	30.5	124	1752.9	30.5	124	1752.9	30.5	124	1752.9
3	54	97	1854.3	74.8	119	3128.1	79.4	118	4251.5
6	72.4	99	3403.6	95.8	98	3867.5	104.8	106	5060
7.5	130.1	82	4048.8	148.5	71	4837.1	164.6	70	5490.1

It can be seen from Fig 12 and Table 5 that the tensile force increases first and then decreases, and the maximum tensile force F_max_ increases with the increase of fiber length. When the length of the three fibers increased from 3cm to 7.5cm, the average value of the maximum tensile force F_max_ was 2.3 times, 3.0 times and 4.8 times that of the non-added fibers, respectively. The maximum tensile force was significantly improved after the fiber was added. With the addition of fiber, the maximum tensile length D_max_ will decrease, and the longer the fiber length, the smaller the D_max_. When the fiber length increased from 3cm to 7.5cm, D_max_ decreased by 10.2%, 18.5% and 40.1%, respectively. This may be because the light components in asphalt are adsorbed by fibers, causing the asphalt to harden. The difference in tensile length D_max_ between fibers of the same length is not significant, indicating that the fiber type has a relatively little effect on D_max_. The yield strain energy W of rubber asphalt is increased by the addition of fiber, and the yield strain energy W increases with the increase of fiber length. When the fiber length increased from 3cm to 7.5cm, the average yield strain energy W was 1.8 times, 2.3 times, and 2.7 times of that without fiber addition. When the fiber length is the same, the rank of the yield strain energy is basalt fiber>alkali free glass fiber>glass fiber. The recommended fiber type is basalt fiber, and the fiber length is 6cm. This is mainly because the fiber will weaken the deformation resistance of the asphalt when it is too long, and it is not easy to disperse during construction.

### 4.2 Fatigue cracking resistance test

The results of fatigue cracking test are shown in Table 6.

**Table 6:** Fatigue life.

Test number	1	2	3	4	5	6	7	8
Fatigue times	7254	9473	9752	10425	12837	14526	16439	15562
Test number	9	10	11	12	13	14	15	16
Fatigue times	16431	18336	16535	17354	19532	18426	16753	14972

**Fig 13:**
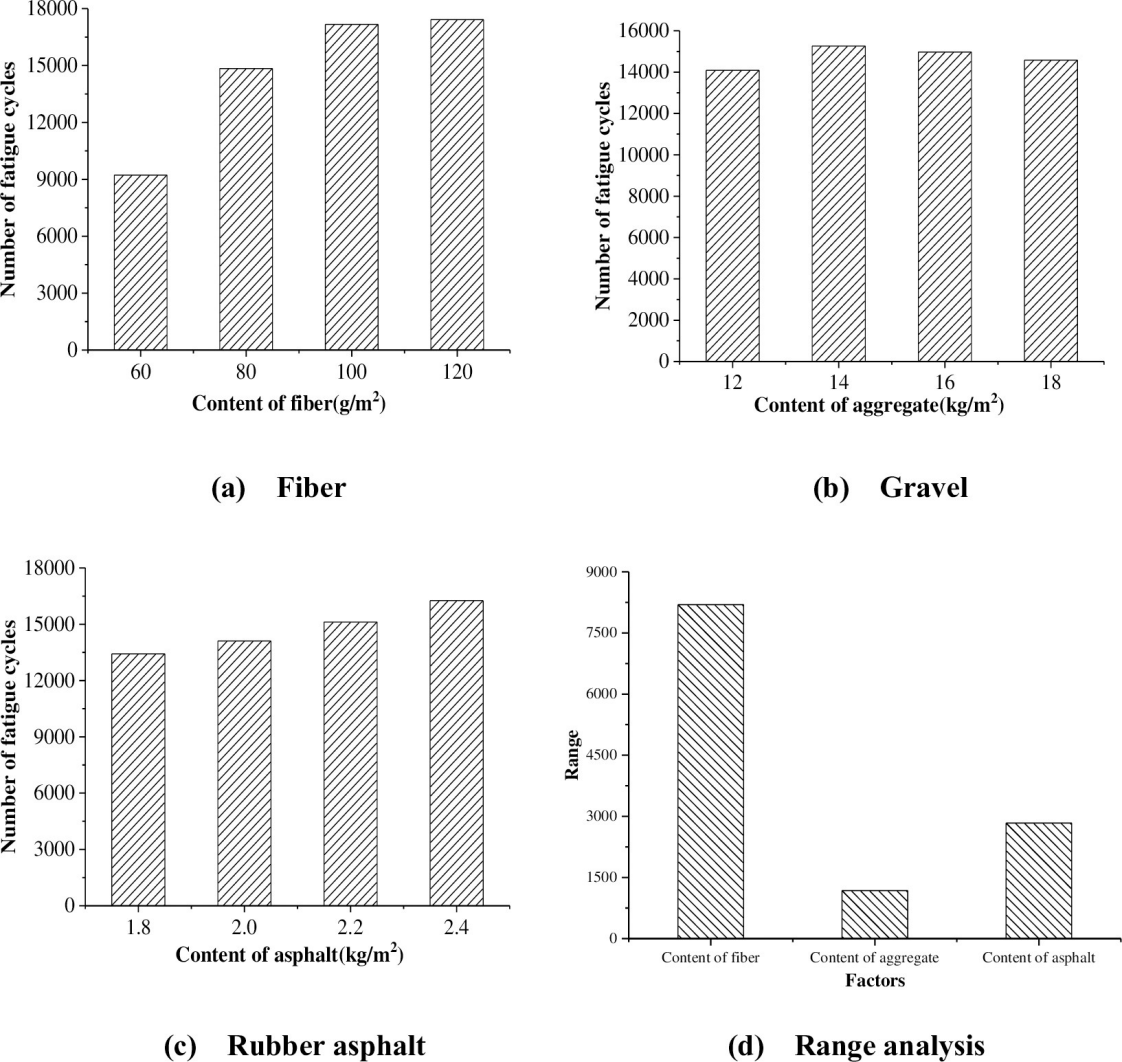
Influence of material dosage on cracking resistance.

Through range analysis, the influence of the amount of fiber, asphalt and gravel on the fatigue times is shown in Fig 13.

It can be seen from Table 6 that the fatigue life of the specimens treated with the fiber rubber asphalt gravel sealing layer are all more than 7000 times, and the maximum value is up to 19,532 times, 2.7 times of the minimum value, indicating that the material ratio has a significant impact on the anti-cracking performance. It can be seen from Fig 13(D) that the amount of fiber has the most obvious influence on the cracking resistance, followed by the amount of asphalt, and finally the amount of gravel. Therefore, to determine a reasonable material ratio is the key to improve the crack resistance. It can be seen from Fig 13(A), with the increase of fiber content, the anti-fatigue cracking ability of the sealing layer becomes better and better. When the fiber dosage was increased from 60g/m^2^ to 80g/m^2^, the crack resistance was increased by more than 50%. When the fiber dosage is increased from 80g/m^2^ to 120g/m^2^, the anti-cracking ability is not much improved, because too much fiber is not easy to disperse and cannot achieve the desired effect. It can be seen from Fig 13(B), the anti-cracking ability of the sealing layer first increases and then decreases with the increase of the amount of gravel, and the anti-cracking ability is the highest when the amount of gravel is 14 kg/m^2^. When the amount of aggregate is too small, the intercalation structure cannot be formed. When the amount of aggregate is too much, it cannot be wrapped by asphalt. Therefore, there is an optimal aggregate amount. It can be seen from Fig 13(C) that the anti-fatigue cracking ability of the sealing layer increases with the increase of the amount of asphalt, but the increase is not obvious. By orthogonal test, the best anti-cracking material combination is A4B2C4, that is, the amount of fiber is 120g/m^2^, the amount of gravel is 14kg/m^2^, and the amount of rubber asphalt is 2.4kg/m^2^.

### 4.3 Fracture energy test

**Fig 14:**
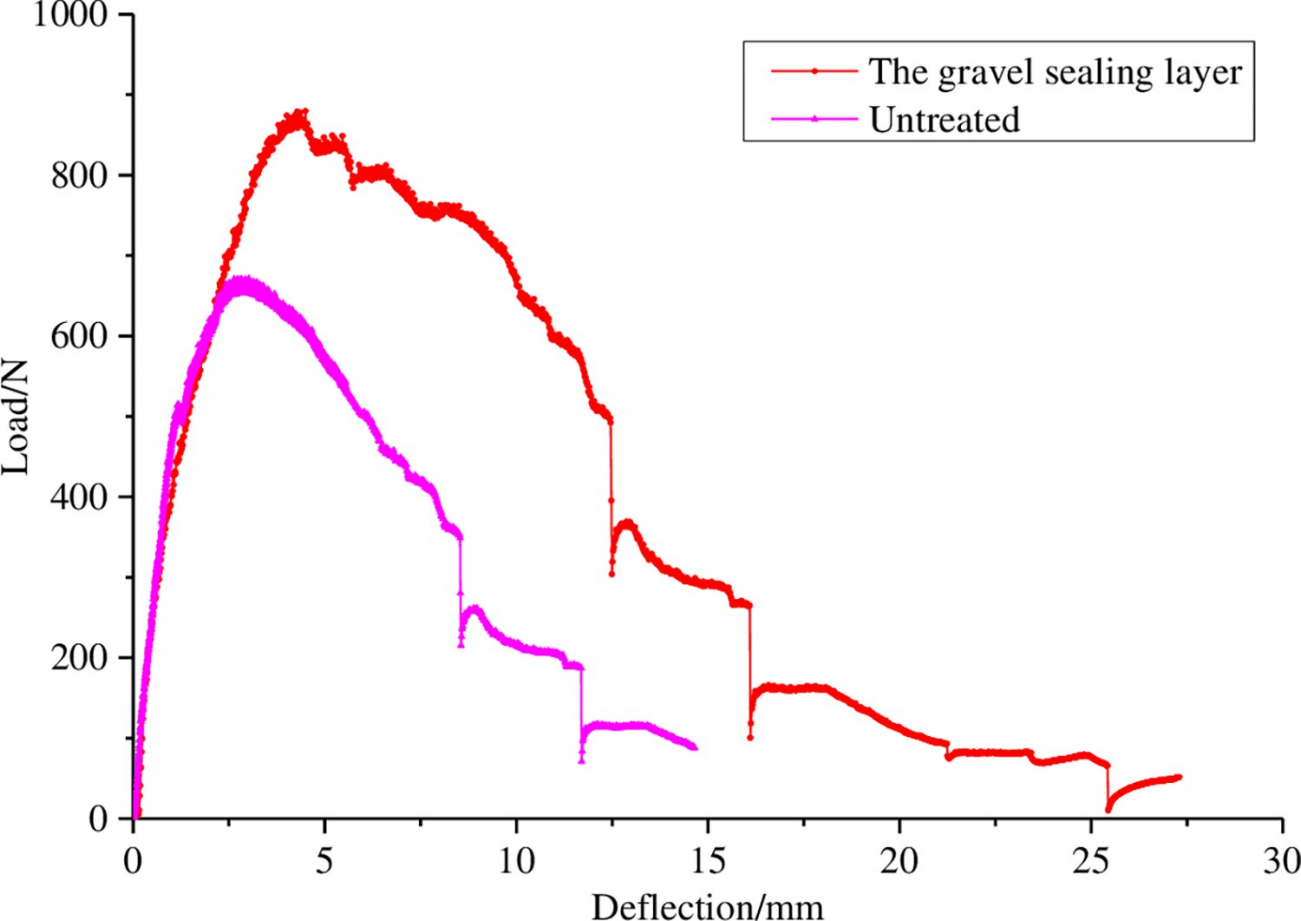
Loading curve of fracture energy test.

The loading curve of fracture energy test for specimens with and without fiber rubber asphalt gravel sealing layer is shown in Fig 14, and the test results are shown in Table 7.

**Table 7:** Results of fracture energy test.

Disposal method	Maximum bending force (N)	Maximum deflection (mm)	Fracture energy (N·mm)
Untreated	671.50	14.64	5318.84
Fiber rubber asphalt gravel seal	879.46	27.29	10609.59

In the experiment, it was observed that the untreated pavement structure first produced a micro-crack near the pre-crack, and then developed upward quickly to form the main crack, which was basically along the vertical direction. The development of pavement cracks with sealing layer is relatively slow, and after the formation of cracks, they first spread along the horizontal direction, and then spread upward, and finally form through the cracks. This is because the gravel can disperse part of the stress concentration, and the fiber asphalt complex increases the toughness of the sealing structure and changes the propagation path of the crack. It can be seen from Fig 14 and Table 7 that the maximum bending force, maximum deflection and fracture energy of the untreated composite pavement structure are 671.50N, 14.64mm and 5318.84N·m, respectively. The maximum bending force, maximum deflection and fracture energy of the pavement structure treated with fiber rubber asphalt gravel sealing layer are 879.46N, 27.29mm and 10609.59N·m, respectively. The maximum bending force is increased by 30.9%, and the maximum deflection and fracture energy are nearly doubled. From the point of view of fracture energy, it can be seen that the crack resistance of the specimen treated with the optimized fiber rubber asphalt gravel sealing layer is obviously improved.

## 5 Conclusion

In order to improve the anti-reflective crack performance of pavement, a fiber rubber asphalt gravel sealing layer with good crack resistance was optimized and designed through the force ductility test, crack fatigue test and fracture energy test. The main conclusions are as follows:

(1) The maximum tension and strain yield energy of rubber asphalt can be significantly increased after the incorporation of fiber, and the strengthening effect is ranked as basalt fiber > alkali-free glass fiber > glass fiber.

(2) The fiber rubber gravel sealing layer has a significant effect on improving the fatigue cracking resistance of the pavement, and the fiber content is the key factor affecting the fatigue life. The optimized material combination with the best crack resistance is 120g/m^2^ fiber, 14kg/m^2^ gravel and 2.4kg/m^2^ rubber asphalt, and the fatigue life can reach 19532 times.

(3) The fracture energy of pavement treated with sealing layer is nearly doubled compared with the untreated pavement, indicating that it has obvious anti-cracking effect.
